# A Rare Case of Spontaneous Closure of Tracheomediastinal Fistula Formed after Chemotherapy for Diffuse Large B-Cell Lymphoma

**DOI:** 10.5761/atcs.cr.26-00047

**Published:** 2026-05-29

**Authors:** Tetsuya Kobayashi, Akie Horikiri, Nanako Yokota, Takafumi Taki, Yoshiaki Takase, Yoshihiko Kanai, Hiroyoshi Tsubochi

**Affiliations:** Department of General Thoracic Surgery, Jichi Medical University, Shimotsuke, Tochigi, Japan

**Keywords:** chemotherapy, diffuse large B-cell lymphoma, spontaneous closure, tracheomediastinal fistula

## Abstract

Tracheomediastinal fistula is a rare but potentially life-threatening complication associated with malignant lymphoma. We report a case of spontaneous closure of a tracheomediastinal fistula that developed after chemotherapy for mediastinal diffuse large B-cell lymphoma (DLBCL). A 70-year-old man presented with airway compression caused by a large mediastinal tumor. After airway stabilization with a Dumon Y stent and subsequent R-CHOP chemotherapy, the tumor markedly regressed. Following stent removal, a fistula developed in the anterior tracheal wall, communicating with a necrotic mediastinal space. Because no clinical or laboratory signs of mediastinitis were observed, conservative management without antibiotics or invasive intervention was adopted. The fistula gradually decreased in size and closed completely within 10 weeks. This case suggests that tracheomediastinal fistulas without mediastinitis may be managed conservatively under careful observation. Treatment strategies should be individualized based on anatomical location and the presence or absence of infection.

## Introduction

Tracheomediastinal fistula is an uncommon complication during the treatment of thoracic malignancies, including lung cancer and lymphoma. When accompanied by mediastinitis, it may lead to severe infection and high mortality, often necessitating surgical repair or airway stenting. However, there are no established guidelines for management, particularly in cases without infection.

We report a rare case of tracheomediastinal fistula that developed during chemotherapy for diffuse large B-cell lymphoma (DLBCL) and subsequently closed spontaneously without invasive intervention.

## Case Report

**Fig. 1:**
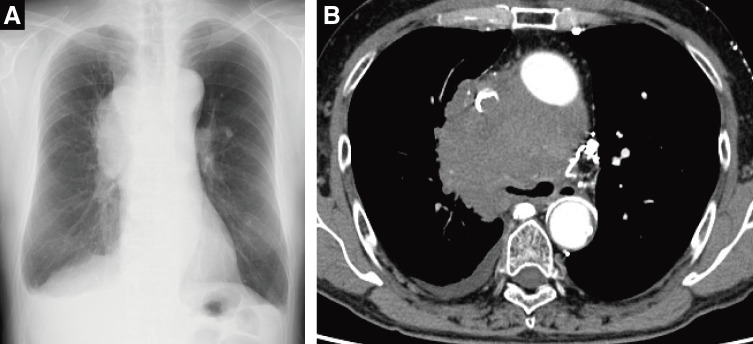
Radiological imaging before treatment. (**A**) A chest radiograph revealed enlargement of the mediastinum and a tumor in the right hilum. (**B**) Contrast-enhanced CT revealed an 11-cm tumor in the mediastinum, compressing the trachea and superior vena cava. CT, computed tomography

**Fig. 2:**
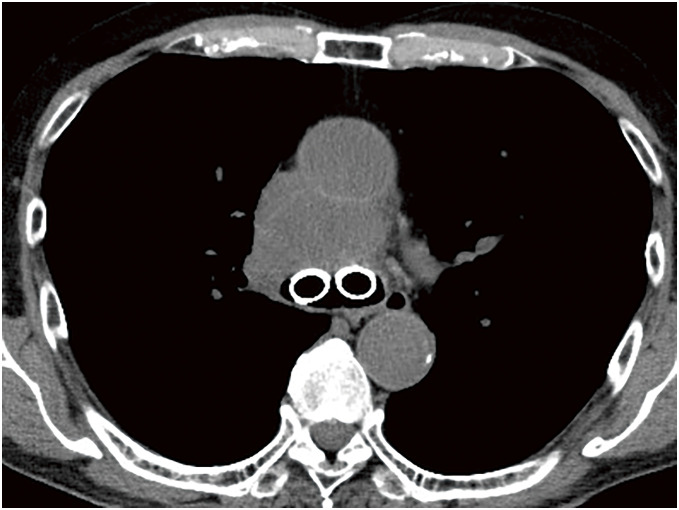
A CT scan taken 65 days after stent placement shows that the tumor has shrunk significantly and the airway stenosis has been resolved. CT, computed tomography

**Fig. 3:**
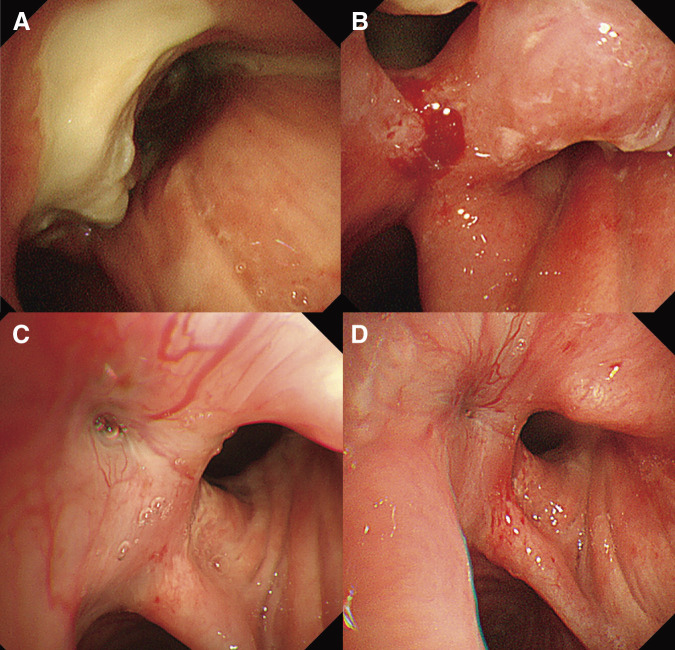
Bronchoscopic findings periodically observed after stent removal. (**A**) After stent removal, necrosis was observed on the anterior wall 1 cm away from the carina. (**B**) Seventeen weeks after stent removal, a fistula was observed in front of the carina. (**C**) Six weeks after the fistula appeared, it had shrunk to a pinhole size. (**D**) Ten weeks after the fistula appeared, it was completely closed.

**Fig. 4:**
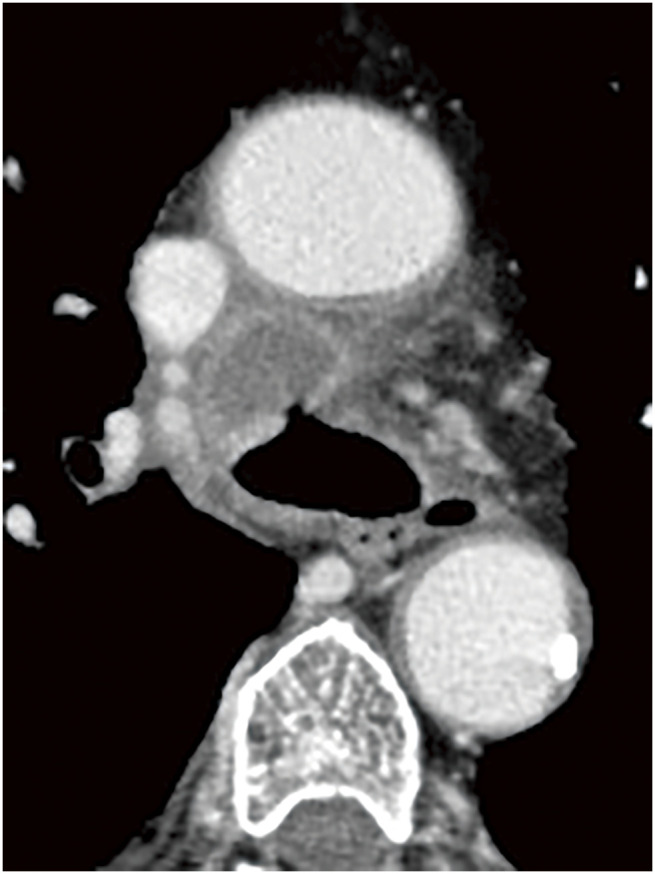
CT images taken at the time of fistula formation showed a low-attenuation area connected to the fistula in front of the carina, which was surrounded by ring-like structures that showed contrast enhancement. CT, computed tomography

**Fig. 5:**
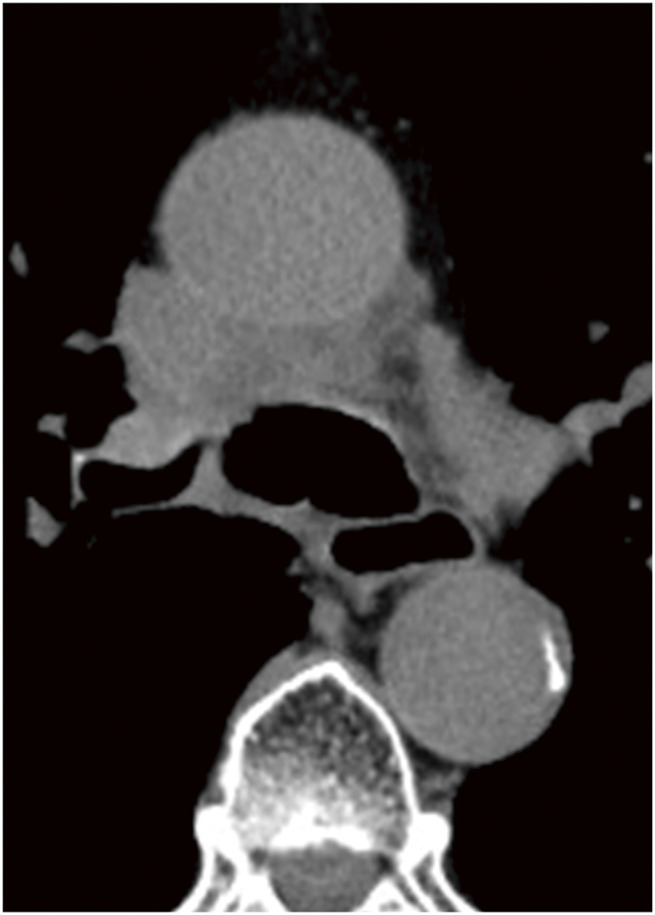
Non-contrast CT image obtained 4 months after the appearance of the fistula, at which time complete closure had been confirmed, showing marked regression of the pretracheal lesion with no evident residual cavity or mass. The status of the previously observed ring-like structure could not be assessed due to the lack of contrast enhancement. CT, computed tomography

A 70-year-old man was referred to our hospital because of a cough and facial edema. Chest radiography revealed a tumor shadow in the right hilum (**Fig. 1A**). Contrast-enhanced computed tomography (CT) revealed an 11-cm tumor extending from the mediastinum to the right hilum (**Fig. 1B**). The tumor was extensively adjacent to the aorta. In addition, the trachea, carina, and right main bronchus were narrowed owing to compression by the tumor. The right pulmonary artery and superior vena cava were also narrowed. Blood tests showed an elevated soluble interleukin 2 receptor (sIL-2R) antibody level (1750 U/mL), while tumor markers, including CEA, CYFRA, ProGRP, and NSE, were within their normal ranges, suggesting lymphoma. Owing to the rapidly progressing respiratory distress, a Dumon Y stent (Novatech, Aubagne, France) was urgently placed under rigid bronchoscopy to secure the airway with the assistance of venovenous extracorporeal membrane oxygenation (ECMO). Bronchoscopy revealed only narrowing without necrosis or fistula in the airway epithelium. After stent placement, the patient was weaned off ECMO and breathing was managed with a ventilator under endotracheal intubation. Three days after stent placement, endoscopic ultrasonography with bronchoscope-guided fine-needle aspiration was performed and methylprednisolone was administered immediately before the diagnosis was confirmed to shrink the tumor. The tumor shrank with methylprednisolone; the tracheal tube was removed on the seventh day after stent placement, and the patient was weaned off the ventilator. The biopsy results showed that the tumor was a DLBCL, and R-CHOP therapy was initiated on the 13th day after stent placement. The tumor gradually shrank and the airway stenosis was relieved (**Fig. 2**); therefore, the stent was removed 10 weeks after placement. At the time of stent removal, necrotic tissue in the anterior tracheal wall was observed 1 cm from the carina (**Fig. 3A**). Flexible bronchoscopy was performed periodically and a 5-mm defect was observed in the mucosa anterior to the carina 17 weeks after stent removal (**Fig. 3B**). The CT scan showed a low-density area in front of the trachea, which was surrounded by a circular structure with some contrast enhancement (**Fig. 4**). Based on these findings, a tracheomediastinal fistula due to tumor necrosis was diagnosed. There was no fever or signs of mediastinitis, such as an increase in white blood cell count or CRP levels; therefore, antibiotics were not administered. Planned radiation therapy for residual lymphoma lesions was not performed, and 2 courses of rituximab were administered to control tumor progression while waiting for the fistula to close. The fistula gradually shrank, and complete closure was confirmed 10 weeks after its appearance (**Figs. 3C** and **3D**). A follow-up non-contrast CT scan obtained 4 months after the appearance of the fistula, at which time complete closure had been confirmed, demonstrated marked regression of the previously observed pretracheal lesion, with no evident residual cavity or mass (**Fig. 5**). Because contrast-enhanced CT was not performed after closure, changes in the previously noted ring-like structure could not be fully evaluated. Intensity modulated radiation therapy (41.4 Gy/ 23 fr) was then administered to the remaining lesions. Six months after treatment initiation, no tumor recurrence was observed. Furthermore, there was no recurrence of the fistula and no complications such as airway stenosis occurred. Written informed consent was obtained from the patient for publication of this case report and accompanying images.

## Discussion

DLBCL originating in the mediastinum grows rapidly and can sometimes cause narrowing of the superior vena cava or trachea. R-CHOP therapy has made it possible to expect a cure in more than 60% of patients with untreated DLBCL.^1,2)^ In localized DLBCL, treatment options include 6–8 cycles of R-CHOP therapy or a combination of 3 cycles of R-CHOP therapy and local radiation therapy.^3)^ In our case, R-CHOP was effective and the tumor shrunk dramatically, but a tracheomediastinal fistula developed during the process.

Tracheomediastinal fistulas can occur in lung cancer and lymphoma; however, there are very few reports on the combination of both tumors. Mediastinitis occurs because of a tracheomediastinal fistula and often becomes severe. Therefore, surgery or stent placement may be performed to prevent the spread of infection.^4–6)^ For example, Tse et al. reported a case of tracheomediastinal fistula that developed in a patient with recurrent Hodgkin’s lymphoma. This case was treated by closing the tracheal defect with a pericardial patch, covering it with omentum, and placing a stent.^4)^ In our case, mediastinitis did not occur, so treatment with rituximab was continued without administration of antibiotics, and the fistula was successfully closed. Radiation therapy has been reported to cause necrosis of the airway,^7)^ raising concerns that it could lead to enlargement of the fistula and worsening of the condition. Therefore, in this case, the planned radiation therapy was not performed, but was administered after confirmation of fistula closure. The factors influencing the occurrence of mediastinitis are unknown. However, in this case, CT scans revealed a membrane-like structure with contrast enhancement around the necrotic tumor, which may have prevented the spread of the infection. In the present case, CT scans revealed a membrane-like structure with contrast enhancement surrounding the necrotic tumor. Although its exact nature remains uncertain, this structure may represent thickened fibrous connective tissue formed during tumor necrosis and subsequent healing in response to effective chemotherapy. Such a structure might have contributed to the containment of the necrotic cavity and the prevention of infection spread. However, because follow-up imaging was limited to non-contrast CT, its post-treatment evolution could not be determined. Huang et al. also reported a case of DLBCL in which a tracheomediastinal fistula developed after chemotherapy, but mediastinitis did not occur, and the fistula healed after proper tumor-specific therapy.^8)^ Mediastinitis caused by a fistula that has developed in the posterior wall of the trachea tends to become severe.^9,10)^ On the other hand, Yamamoto et al. reviewed tracheomediastinal fistulas in patients with lung cancer and lymphoma and reported that fistulas originating in the anterior or lateral walls had a better prognosis than those originating in the posterior wall.^11)^ Our case also involved a fistula in the anterior wall of the trachea; it may be that the anatomical background made it difficult for the inflammation to spread. In our case, we did not administer antibiotics because there was no concomitant mediastinitis. However, mediastinitis can cause serious conditions; therefore, careful observation is necessary to detect its onset as early as possible.

In summary, we experienced a case of tracheomediastinal fistula that developed after R-CHOP therapy in a patient with DLBCL. As there was no mediastinitis, no antibiotics were administered, and rituximab was continued, resulting in closure of the fistula. As tracheomediastinal fistulas without mediastinitis may close spontaneously, invasive treatments such as surgery or stent placement may not always be necessary in such cases, and their indications should be carefully considered.

## Declarations

### Data availability

No new data were created or analyzed in this study. Data sharing is not applicable to this article.
